# Assessment of survival outcomes among lung cancer patients at the National and Referral Hospital in Kenya

**DOI:** 10.1002/cam4.5658

**Published:** 2023-01-27

**Authors:** Nur Swaleh Said, Amsalu Degu

**Affiliations:** ^1^ Department of Pharmaceutics and Pharmacy Practice School of Pharmacy and Health Sciences, United States International University‐Africa Nairobi Kenya

**Keywords:** Kenya, Kenyatta National Hospital, lung cancer, survival outcomes

## Abstract

**Introduction:**

Lung cancer has a low overall survival rate linked to late diagnosis and metastasis. Unfortunately, comprehensive data within the African continent are limited due to the lack of a registry, low public awareness of lung cancer, financial constraints, and inadequate screening and treatment facilities. In addition, there was a lack of conclusive data in our setting. Therefore, this study aimed to assess survival outcomes among lung cancer patients at Kenyatta National Hospital.

**Methods:**

A hospital‐based retrospective cohort study was performed to examine the survival outcomes of 151 lung cancer patients. All eligible lung cancer patients diagnosed and treated in the facility between January 1, 2018, and December 31, 2020, were included. The patients were retrospectively followed from the date of primary cancer diagnosis until death or the last follow‐up period. The Statistical Package for the Social Sciences (SPSS) version 20.0 statistical software was used to enter and analyze the data. Kaplan–Meier survival and Cox regression analysis were employed to determine median survival and predictors of mortality, respectively.

**Results:**

The mean and median follow‐time was 18.2 and 17.5 months, respectively. Most (98%) of the patients had non‐small cell lung cancer. The 2‐year survival rate was 66.7%, with 59.6% of patients having developed distant metastasis during the follow‐up, while 25.1% were deceased. The median cancer‐specific survival time among the study population was 18.0 ± 3.40 months. Cox regression analyses showed that patients with distant metastasis had five times more risk of dying (AHR: 4.74, 95% CI: 2.1–10.8, *p* < 0.001) than patients without distant metastasis.

**Conclusions:**

The overall two‐year survival rate of lung cancer patients at the Kenyatta National Hospital was 66.7%, with most patients developed distant metastasis during the follow‐up period. Distant metastasis was the only significant predictor of mortality among lung cancer patients in our setting.

## Graphical abstract

The prognosis of lung cancer is still poor despite several treatment strategies. A retrospective cohort studywas employed among 151 adult lung cancer patients to assess survival outcomes. The study revealed 75.5% one‐year and 66.7% two‐year survival rates, with most patients having distant metastasis in the follow‐up period.

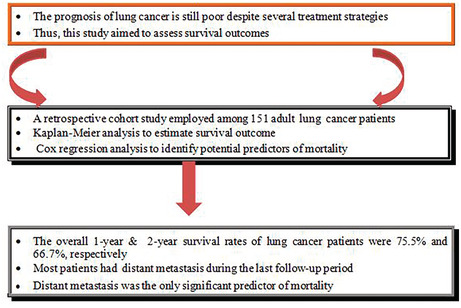

## 1 INTRODUCTION

Lung cancer is the leading cause of cancer‐related death, accounting for an estimated 1.8 million fatalities globally.^1^ It continues to be a major global public health issue, necessitating the deployment of scientific and effective control strategies.^2^ In America and Europe, new lung cancer cases constituted 13% and 11.8% of all recorded cancers, respectively.^1^ Lung cancer was also reported to have the highest mortality rate, corresponding to one‐fifth of the overall share of cancer‐related deaths.^3^ In 2020, Globocan estimated 1,435,943 incidences of lung cancer, out of which 794 cases were from Kenya.^1^ South and North African countries have a greater prevalence of lung cancer than East and West Africa.^4^ This malignancy is known to have a poor survival rate, but an improvement in the overall survival rates can be attributed to personalized treatment and the introduction of targeted therapy.^5^ In a Scandinavian study, an improvement in 1‐year survival was observed in nonsquamous cell carcinoma regardless of the stage of the malignancy at diagnosis. However, only those with stages I and II of the malignancy showed a longer‐term 5‐year survival.^6^ Previous studies have reported poor overall survival among lung cancer patients.^7^, ^8^ Several factors associated with the survival outcomes of lung cancer, including age, gender, performance status, detailed tumor location, histological type, and initial treatment modality, are crucial predictive variables in the survival of lung cancer patients.^9^, ^10^ Inadequate management and underdiagnosis of lung cancer are alarming issues in the health care of African settings.^4^, ^10^ Access to cancer screening and treatment is one of the major challenges in Kenya.^11^

Furthermore, previous studies reported the high cost of treatment, inadequate knowledge about cancer, poor health‐seeking behavior and long distance to access cancer treatment facilities were the key barriers to optimal cancer treatment in the local setting.^12^ This can compromise the survival of patients with lung cancer. Despite several studies reporting poor survival outcomes in lung cancer, there was a lack of conclusive data about the survival outcomes of lung cancer patients in our setting. Therefore, the study aimed to assess survival outcomes among lung cancer patients at Kenyatta National Hospital.

## 2 MATERIALS AND METHODS

### 2.1 Study design, setting, and period

A retrospective cohort study was conducted among lung cancer patients at Kenyatta National Hospital (KNH) from May 1 to July 31, 2022. KNH is a Level 6 hospital that serves as the national referral and teaching public hospital. It is Kenya's leading referral hospital situated on the hospital road along Upper Hill, Nairobi city. The hospital has 2000 bed capacity and mainly treats referral cases from different parts of the country. However, there is no restriction only to referral cases in order to get treatment in the study setting. Three major hospitals, including Mediheal Group of Hospitals, Upper Hill Medical Center, and the German Medical Center, are located within a few kilometers from KNH. This study design was chosen since survival outcome takes a long time to note after introducing various cancer treatment modalities and requires intensive follow‐up. Accordingly, reliable determination of the survival outcomes several years after treatment commencement could still be done retrospectively.

### 2.2 Study population

The study population included all eligible lung cancer patients diagnosed and treated at Kenyatta National Hospital's Oncology Department from January 1, 2018, to December 31, 2020.

### 2.3 Eligibility criteria

#### 2.3.1 Exclusion criteria

Patients having gaps in their medical records on their diagnosis, histology, and treatment plan were excluded from the study.

#### 2.3.2 Sample size

According to the Health Information and Records Department of KNH, 221 new lung cancer cases were diagnosed and treated in the study setting over 3 years (January 1, 2018, to December 31, 2020). Out of 221 medical records, medical records of 151 lung cancer patients met the eligibility criteria and were included in the study.

#### 2.3.3 Inclusion criteria

All adult patients (≥18 years) with a confirmed diagnosis of lung cancer, had complete medical records of diagnosis, histological classification, and treatment plan, and received at least one form of lung cancer treatment in the study setting were included in the study. To be included in the study, the patient must have been diagnosed and treated in the facility from January 1, 2018, to December 31, 2020.

#### 2.3.4 Research instrument and data collection procedures

A data abstraction tool was designed after reviewing previous studies.^10^, ^13^, ^14^ The data abstraction tool was composed of sociodemographic characteristics, clinical characteristics, and treatment outcomes measuring parameters such as mortality, median cancer‐specific survival, year of survival, and status of metastasis.

Oncology nurses and pharmacists were involved in the data collection after training. A pretest was conducted on 5% of the total sample size. Before using them in the main study, all essential improvements were made in the data abstraction tool. All medical records of lung cancer patients diagnosed and treated from January 1, 2018, to December 31, 2020 were sourced from the hospital's Health Information and Record Department using their hospital patient identification numbers. Then, the data collectors screened medical records of all 221 lung cancer patients to assess their eligibility for the study using the predefined criteria. After that, the medical records of all eligible 151 lung cancer patients were retrospectively reviewed from the date of primary diagnosis to the last follow‐up or death. Concurrent comorbidities were also recorded based on the documented information of medical records of the patients. The median cancer‐specific survival time was computed by calculating the time from the primary lung cancer diagnosis date until the last follow‐up period in the study setting (last visit) or death using the documented information from the medical records of the patients. Mortality was also assessed using the documented medical information of the patients, such as a death summary report issued by the hospital. The mortality rate was estimated by dividing the total number of deaths during the follow‐up period by the total sample population enrolled in the study. The 1‐year survival rate was calculated by dividing the total number of lung cancer patients who survived 1 year after the primary cancer diagnosis by the total number of patients at risk of dying in the same year and multiplying by 100. The 2‐year survival rate was computed by first estimating the survival probability in Year 2. This was calculated by dividing the total number of lung cancer patients who survived 2 years after the primary cancer diagnosis by the total number of patients at risk of dying in the same year. After that, the 2‐year survival rate was computed by multiplying the survival probability of Year 1 and Year 2 by 100. Unfortunately, it is impossible to compute the 3 years survival rate in this study since we do not have adequate documentation of follow‐up periods up to 3 years to compute the three‐year survival rate of lung cancer patients. The presence of distant metastasis was assessed based on the findings of the interval scans done for each patient during the follow‐up period.

#### 2.3.5 Data analysis

The program Statistical Package for the Social Sciences (SPSS) version 20.0 was used to enter and analyze the data. Kaplan–Meier analysis was used to analyze the median survival of patients. Cox proportional hazards regression analysis was used to examine the association between independent variables and the risk of death. Variables with a *p*‐value <0.2 in the bivariate analysis were included in the multivariate analysis. The variables used were age, gender, comorbidity, distance metastasis, and the type of treatment involved. Percentages and frequencies were employed for data presentation.

## 3 RESULTS

### 3.1 Sociodemographic characteristics of patients

From January 1, 2018, to December 31, 2020, 221 lung cancer patients were diagnosed in the study setting. The median age of diagnosis of lung cancer patients was 63.0 ± 14.7 years. The mean and median follow‐time were 18.2 and 17.5 months, respectively. More than half (68.2%) of the study population were males, while 31.8% were females. Regarding education status, 31.8% of the patients reached a primary level, 19.9% reached a secondary level, 9.3% achieved a tertiary level, 13.2% were illiterate, and 25.9% had an undocumented status (Table 1).

**TABLE 1:** Sociodemographic characteristics of the study participants.

Variables	Frequency	Percent (%)
Age		
≥60 years	82	54.3
<60 years	66	43.7
Gender		
Male	103	68.2
Female	48	31.8
Marital status		
Single	12	7.9
Married	132	87.4
Divorced	2	1.3
Widowed	4	2.6
Educational status		
Primary	48	31.8
Secondary	30	19.9
Tertiary	14	9.3
Illiterate	20	13.2
Unknown	39	25.9
History of substance use		
Smoking cigarette	15	9.9
Alcohol	1	0.7
Unknown status	117	77.5
Nonsubstance users	18	11.9

### 3.2 Clinical characteristics of the study participants

Histologically, 98% of the patients had non‐small cell lung carcinoma, while 2.0% of the patient's histological classification was not specified. Retroviral disease was the most prevalent comorbidity, accounting for 18% of all patients (Table 2).

**TABLE 2:** Clinical characteristics of lung cancer patients.

Variable	Frequency	Percent (%)
Histological type of cancer		
Non‐small cell lung cancer	148	98
Unspecified	3	2
Comorbidity		
Present	38	25.2
Absent	110	72.8
Unknown	3	2
Types of comorbidity		
Retroviral diseases	7	18
Pneumonia	4	10.3
Diabetes type 2	4	10.3
Pleural effusion	4	10.4
Malignant pleural effusion	3	7.7
Convulsions	3	7.7
Hypertension	2	5.1
Brain metastasis	2	5.1
Sickle cell disease	1	2.6
Tuberculosis	1	2.6
Supraglottic tumor	1	2.6
Anemia	1	2.6
Bronchogenic carcinoma	1	2.6
Acute kidney injury	1	2.6
Melanoma	1	2.6
Arthritis	1	2.6
Bilateral neural effusion	1	2.6
Renal tubular acidosis	1	2.6

Most patients (44.4%) underwent chemotherapy as their standard treatment for lung cancer, followed by both radiotherapy and chemotherapy (25.2%). In addition, most patients underwent five to eight cycles of chemotherapy to manage their condition (Table 3).

**TABLE 3:** Treatment regimen of the study participants.

Variable	Frequency	Percentage (%)
Treatment regimen		
Chemotherapy	67	44.4
Radiotherapy + chemotherapy	38	25.2
Radiotherapy	31	20.5
Surgery	10	6.6
Surgery + chemotherapy	5	3.3
Type of chemotherapy		
Carboplatin + paclitaxel	21	13.9
Cisplatin + etoposide	17	11.3
Cisplatin + paclitaxel	13	8.6
Carboplatin + docetaxel	8	5.3
Cisplatin + docetaxel	7	4.6
Gemcitabine + vinorelbine	2	1.3
Docetaxel + cisplatin + 5‐fluorouracil	1	0.7
Geftinib	1	0.7
Number of cycles		
1–2	15	22.3
3–4	22	32.8
5–8	33	49.3
Prophylactic antiemetic regimen		
Yes	30	19.9
No	118	78.1
Unknown	3	2.0

### 3.3 Treatment outcomes of lung cancer patients

A predominant number of patients (59.6%) had evidence of distant metastasis in the last follow‐up period, while 40.4% (61) had no distant metastasis. The mortality rate among lung cancer patients was 25.1%. The median cancer‐specific survival time among the patient was 18.0 ± 3.40 months. According to the current study, lung cancer patients had a 1‐year and 2‐year survival rate of 75.5% and 66.7%, respectively.

### 3.4 Predictors of mortality among lung cancer patients

Cox regression analyses showed that patients with distant metastasis had five times more risk of dying (AHR: 4.74, 95% CI: 2.1–10.8, *p* < 0.001) than patients without distant metastasis. Nonetheless, gender, age, comorbidity, and treatment regimens were not statistically significant predictors of mortality among lung cancer patients (Table 4).

**TABLE 4:** Predictors of mortality among lung cancer patients.

Variable	Bivariate analysis		Multivariate analysis	
	CHR (95% CI)	*p*‐Value	AHR (95% CI)	*p*‐Value
Age (in years)				
<60 years	1.376 (1–2.0)	0.055	1.379 (0.7–2.8)	0.368
≥60 years	1	1	1	
Gender				
Male	1.694 (0.8–3.6)	0.174	1.353 (0.6–2.9)	0.453
Female	1	1	1	
Distant metastasis				
Yes	6.2 (2.8–13.5)	<0.001*	4.74 (2.1–10.8)	<0.001*
No	1	1	1	
Comorbidity				
Present	3.9 (1.2–12.8)	0.023*	2.6 (0.8–8.9)	0.113
Absent	1	1	1	
Type of treatment				
Chemotherapy	1.038 (0.5–2.0)	0.21	2.503 (0.3–23)	0.423
Radiotherapy + chemotherapy	0.382 (0.2–0.9)	0.021*	1.116 (0.1‐11.8)	0.927
Radiotherapy	2.64 (0.76–6.1)	0.005*	6.234 (0.6‐62)	0.119
Surgery	2.151 (0.8–6.1)	0.091	4.867 (0.4–58.5)	0.213
Surgery + chemotherapy	1	1	1	1

Abbreviations: AHR, adjusted hazard ratio; CHR, crude hazard ratio.

* Statistically significant with *p*‐value <0.05.

## 4 DISCUSSION

This retrospective study purposed to analyze the survival outcomes of lung cancer patients treated at Kenyatta National Hospital. In 2020, lung cancer accounted for about one in 10 (11.4%) new cases of cancer and one in five (18.0%) fatalities, making it the second most prevalent neoplastic disease diagnosed and the leading cause of cancer mortality.^1^

The present study showed that lung cancer patients' 1‐year and 2‐year survival rates were 75.5% and 66.7%, respectively. In contrast, the Ugandan study demonstrated lower 1‐year (41.7%) and 2‐year (29.7%) survival rates.^7^ An Iranian study also reported a lower rate of 1‐year (39%) and 2‐year (18%) survival of lung cancer patients.^15^ The disparity in the survival outcomes between our study and those of other studies is probably linked to the difference in the age of the study population, stage of the disease, comorbidity, duration of the follow‐up period, and histological type of cancer. Therefore, early screening programs and optimal management of the patients are indispensable for better survival among lung cancer patients. However, a Chinese study showed an overall increased survival over time among non‐small cell lung cancer patients^16^ attributable to personalized treatment and the introduction of targeted chemotherapy.

The findings of our study revealed that the majority (68.2%) of the patients were males, while 31.8% were females. According to a similar study, 62.4% of the patients were male, and 37.6% were female.^17^ Other studies in the Middle East and African countries also reported a male predominance of lung cancer cases.^18^ The disease exhibits predominance in the male population compared with females. This could be attributed to the history of smoking which accounts for more men smoking tobacco than women, increasing the incidence and mortality rates.^19^

In the study, most lung cancer patients were 60 years of age and above (54.3%), with a median age of 63 years. In New Zealand, the median age of diagnosis is 71 years.^20^ China reported a dramatic increase in lung cancer incidence and mortality in the population above 60 years, making it clear that the incidence and mortality in men and women increase with age in their population.^21^ The results can be attributed to the structural, physiological, and immunological changes that occur with age.

Patients with comorbidities made up roughly a quarter of the total population, with human immunodeficiency virus (HIV) being the most common type of comorbidity (18%). The primary cause of cancer‐related death and a significant cause of morbidity among Americans with HIV infection is lung cancer.^22^ However, Leduc et al. reported that the high risk of lung cancer in HIV‐positive patients is a multifactorial concept. But, immunodeficiency, especially CD4 lymphocyte cell count, is the most significant predicted risk factor for cancer in HIV patients.^23^

Non‐small cell lung cancer is the most common histological type of lung cancer in many areas.^24^ In Europe, non‐small cell lung cancer represents 85%–90% of all lung cancer cases.^25^ John et al. found most cases of lung cancer in Australia to be of non‐small cell lung cancer histopathology, making it prevalent in 64% of the male population and 61% of the female population diagnosed with lung cancer.^26^ In addition, the Ugandan study also showed that most lung cancer patients (96.6%) had non‐small cell lung cancer.^7^ Despite our study constituting a smaller sample size, we found 98% of the lung cancer cases to be of non‐small cell lung cancer histopathology.

The recommended treatment regimen for patients with unresectable lung cancer is radiotherapy with concurrent chemotherapy. The West Japan Thoracic Oncology Group was the first to show that concurrent chemoradiation enhanced response and survival rate much more than sequential chemoradiation.^27^ Only a quarter of our study population was treated with radiotherapy and chemotherapy, which could be attributed to radiotherapy not being readily available to most patients. The most common chemotherapy regimen used was carboplatin + paclitaxel. The standard guideline also recommends the combination of platinum compounds (carboplatin or cisplatin) with a taxane (paclitaxel).^24^ Nonetheless, there is no agreement on which chemotherapy is better in conjunction with radiotherapy.^28^

The cancer‐specific survival time among the patients in the study was 18.0 ± 3.40 months. A study conducted in China estimated the median relative survival to have improved from 8.69 months to 10.38 months,^16^ while another in Turkey revealed a median survival of 11.77 ± 1.00 months in all patients diagnosed with non‐small cell lung cancer.^29^

Age, gender, performance status, tumor location, histological type, and initial treatment modalities were significant predictors of survival in stages I and II lung cancer patients.^9^ In the advanced stages, age, performance status, tumor laterality, tumor location, and initial treatment modality were the significant predictors of survival.^30^ Nonetheless, distant metastasis was the only significant factor affecting survival outcomes among lung cancer patients in our setting.

### 4.1 Limitations of the study

The lack of proper documentation of medical records in the setting can affect the generalizability of the study findings. In addition, incomplete patient files reduced the sample size pool, limiting the study's sample population.

## 5 CONCLUSIONS

The overall 1‐year and 2‐year survival rates of lung cancer patients were 75.5% and 66.7%, respectively, with most patients having distant metastasis during their last follow‐up period. Cox regression analyses showed that patients with distant metastasis had five times more risk of dying than patients without distant metastasis. Early screening programs and optimal management of the patients are indispensable for better survival among lung cancer patients in our setting.

## ETHICS APPROVAL

After receiving approval from the University of Nairobi/Kenyatta National Hospital Ethics and Research Committee (Approval number: UP142/02/2022), the actual data collection was carried out. The confidentiality and anonymity of all patient information and their records were maintained. Patient records remained on the premises, and the information gathered was only used for the purpose intended.

## PATIENT CONSENT

Due to the retrospective nature of the study, informed consent waivers from the Ethics committee were obtained.

## AUTHOR CONTRIBUTIONS

**Nur Swaleh Said:** Conceptualization (equal); data curation (equal); formal analysis (equal); funding acquisition (equal); investigation (equal); methodology (equal); project administration (equal); resources (equal); software (equal); supervision (equal); validation (equal); visualization (equal); writing – original draft (equal); writing – review and editing (equal). **Amsalu Degu:** Conceptualization (equal); data curation (equal); formal analysis (equal); funding acquisition (equal); investigation (equal); methodology (equal); project administration (equal); resources (equal); software (equal); supervision (equal); validation (equal); visualization (equal); writing – original draft (equal); writing – review and editing (equal).

## FUNDING INFORMATION

There was no funding to conduct this study.

## CONFLICT OF INTEREST

The authors state that they do not have any conflicts of interest.
